# Opportunities to accelerate extracellular vesicle research with cell‐free synthetic biology

**DOI:** 10.1002/jex2.90

**Published:** 2023-05-18

**Authors:** Richard J. R. Kelwick, Alexander J. Webb, Amelie Heliot, Clara Tresserras Segura, Paul S. Freemont

**Affiliations:** ^1^ Section of Structural and Synthetic Biology Department of Infectious Disease Imperial College London London UK; ^2^ Department of Metabolism Digestion and Reproduction, Imperial College London London UK; ^3^ The London Biofoundry Imperial College Translation & Innovation Hub London UK; ^4^ UK Dementia Research Institute Care Research and Technology Centre Imperial College London, Hammersmith Campus London UK

**Keywords:** cell‐free synthetic biology, diagnostics, EV mimetics, exosomes, extracellular vesicles, therapeutics

## Abstract

Extracellular vesicles (EVs) are lipid‐membrane nanoparticles that are shed or secreted by many different cell types. The EV research community has rapidly expanded in recent years and is leading efforts to deepen our understanding of EV biological functions in human physiology and pathology. These insights are also providing a foundation on which future EV‐based diagnostics and therapeutics are poised to positively impact human health. However, current limitations in our understanding of EV heterogeneity, cargo loading mechanisms and the nascent development of EV metrology are all areas that have been identified as important scientific challenges. The field of synthetic biology is also contending with the challenge of understanding biological complexity as it seeks to combine multidisciplinary scientific knowledge with engineering principles, to build useful and robust biotechnologies in a responsible manner. Within this context, cell‐free systems have emerged as a powerful suite of *in vitro* biotechnologies that can be employed to interrogate fundamental biological mechanisms, including the study of aspects of EV biogenesis, or to act as a platform technology for medical biosensors and therapeutic biomanufacturing. Cell‐free gene expression (CFE) systems also enable *in vitro* protein production, including membrane proteins, and could conceivably be exploited to rationally engineer, or manufacture, EVs loaded with bespoke molecular cargoes for use in foundational or translational EV research. Our pilot data herein, also demonstrates the feasibility of cell‐free EV engineering. In this perspective, we discuss the opportunities and challenges for accelerating EV research and healthcare applications with cell‐free synthetic biology.

## 1 INTRODUCTION

**FIGURE 1:**
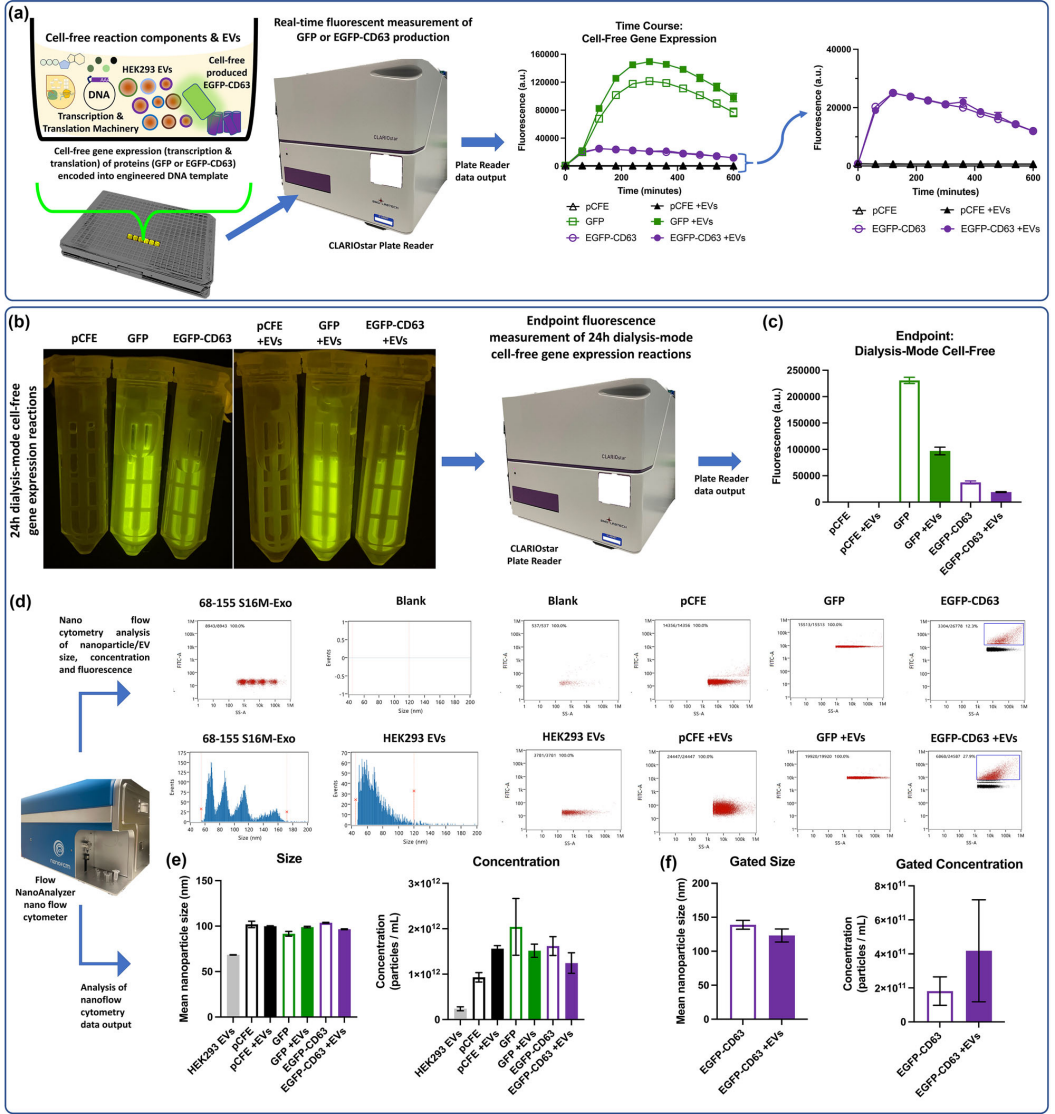
Cell‐free gene expression‐based extracellular vesicle engineering. (a) Time course analysis of GFP or EGFP‐CD63 protein production in 10 μL‐scale cell‐free gene expression reactions with (+EVs) or without the inclusion of HEK293‐derived extracellular vesicles. pCFE denotes control cell‐free reactions that used empty vector DNA. *n* = 8 independent cell‐free batch reactions. (b) Representative photo of 100 μL‐scale ‘semi‐continuous dialysis mode’ cell‐free gene expression reactions. Samples were placed on a BluPAD LED transilluminator (Bio‐Helix) and imaged. (c) Endpoint (24 h) analysis of GFP or EGFP‐CD63 protein production in 100 μL‐scale ‘semi‐continuous dialysis mode’ cell‐free gene expression reactions with (+EVs) or without the inclusion of HEK293‐derived extracellular vesicles. pCFE denotes control empty vector cell‐free reactions. *n* = 3 independent dialysis‐mode cell‐free reactions. (d) Representative size histograms or dot plots of size reference standard silica nanospheres (68–155 nm S16M‐Exo), blank (HEPES/NaCl buffer), HEK293 extracellular vesicles or indicated ‘dialysis‐mode’ cell‐free gene expression reactions (pCFE, GFP or EGFP‐CD63). The inclusion of HEK293 cell‐derived EVs within the indicated cell‐free reaction is denoted with +EVs. (e) Mean nanoparticle size and concentration quantification of HEK293 extracellular vesicles and of nanoparticles within the indicated ‘semi‐continuous dialysis mode’ cell‐free gene expression reactions. n = 3 independent dialysis‐mode cell‐free reactions. (f) Mean nanoparticle size and concentration quantification of gated nanoparticle sub‐population, as indicated by the blue gate boxes in this figure panel d (EGFP‐CD63 and EGFP‐CD63 +EV cell‐free gene expression reactions). Error bars denote standard error of the mean, *n* = 3 independent dialysis‐mode cell‐free reactions.

**FIGURE 2:**
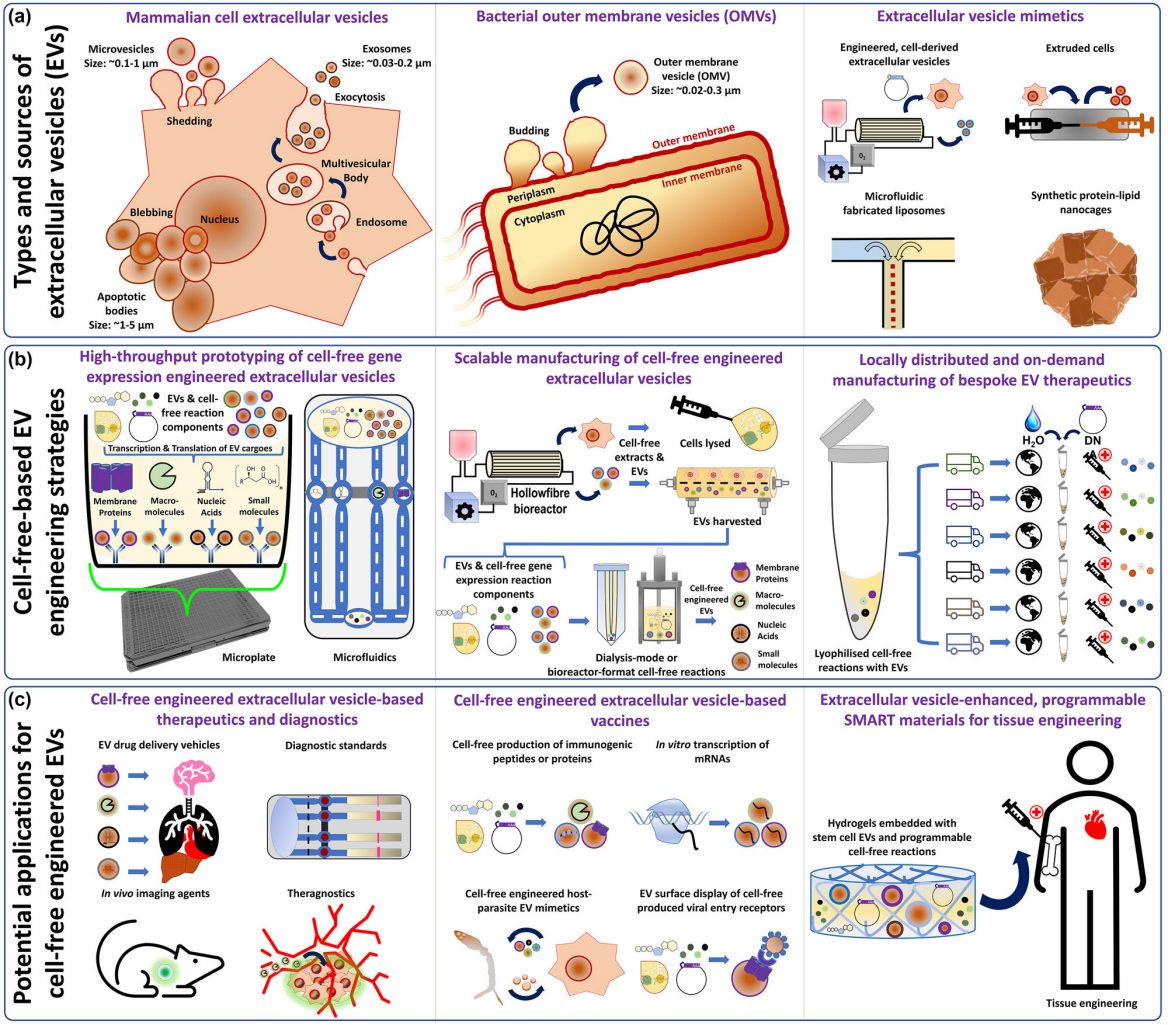
Cell‐free extracellular vesicle engineering strategies and future medical applications. (a) Extracellular vesicle (EV) types and sources including exosomes, microvesicles, apoptotic bodies, bacterial outer membrane vesicles (OMVs) and EV mimetics (including engineered EVs from genetically engineered cells, EVs derived from extruded cells, microfluidic fabricated lipid nanoparticles and protein nanocages). (b) Cell‐free gene expression strategies for extracellular vesicle engineering—including plate or microfluidic cell‐free reaction formats to enable the rapid prototyping of many EV‐cargoes, dialysis‐mode or bioreactor format cell‐free reactions for scalable EV engineering or a distributed manufacturing format in which lyophilised cell‐free reactions are stored ready for on‐demand cell‐free manufacturing of EV‐based therapeutics or vaccines. (c) Potential downstream applications for cell‐free engineered extracellular vesicles include therapeutic drug delivery, diagnostics, theragnostic approaches, EV‐based vaccines and tissue engineering.

Cell‐free synthetic biology encompasses many different *in vitro* biotechnologies that utilise isolated biological machinery or cellular biochemical processes for useful purposes. Whilst many different types of cell‐free systems have been developed, cell‐free gene expression (CFE) is an important application area that enables *in vitro* protein production from DNA templates. CFE systems typically make use of transcription and translation machinery within processed cell extracts (lysates) or purified recombinant cellular machinery as their core mechanism. Cell‐free synthetic biology has diverse applications including metabolic engineering, biological materials, environmental monitoring, medical diagnostics and therapeutic biomanufacturing (Garenne et al., 2021; Kelwick et al., 2020; Monck et al., 2022; Swartz, 2018; Zawada et al., 2022). Cell‐free synthetic biology may also be poised to enable a radical biomanufacturing paradigm, whereby therapeutic and medical diagnostic technologies can be readily manufactured locally from rapidly designed and *in silico* distributed genetic sequences (Ogonah et al., 2017; Tinafar et al., 2022). Importantly, cell‐free reactions can be lyophilised or silica dried and potentially employed in areas with limited cold chain access (Guzman‐Chavez et al., 2022; Jaenes et al., 2022; Pardee, 2018; Pardee et al., 2014). In this perspective article, we envision future convergences between cell‐free synthetic biology and extracellular vesicle (EV) research, which may create new opportunities for medical innovations. Indeed, EVs, including exosomes, are being explored for their diagnostic or therapeutic potential across a broad array of chronic health conditions and infectious diseases (De Sousa et al., 2022; Kalluri & Lebleu, 2020; Pearson et al., 2021; Roy et al., 2018). Exosomes are nano‐sized EVs, of endosomal origin, that are secreted by most eukaryotic cell types (Couch et al., 2021; Kalluri & Lebleu, 2020). Interestingly, aspects of exosome biogenesis have also been studied *in vitro*, including the use of cell‐free biochemical assays to delineate important molecular mechanisms of miRNA sorting into HEK293T exosomes (Shurtleff et al., 2016). As such, cell‐free systems can be employed to study and understand fundamental aspects of exosome biology. The development of EV mimetics, such as those generated through cell extrusion or directly from bespoke lipid compositions, is also rapidly progressing and somewhat benefits from efforts towards cell‐free‐based minimal cell engineering (Garamella et al., 2016; Jia et al., 2021; Sato et al., 2016; Staufer et al., 2021). Likewise, cell‐free synthetic biology methods have been established for *in vitro* membrane protein production and subsequent incorporation within lipid structures (e.g., liposomes or nanodiscs) (Bruni et al., 2022; Shelby et al., 2019). Such approaches could conceivably be repurposed to generate membrane fusion proteins or other cargoes for the purposes of engineering and functionalising cell derived or synthetic EVs. Our pilot data herein also demonstrates the feasibility of cell‐free EV engineering (Figure 1; Supplementary Figures). From our perspective, these developments provide a direction towards more sophisticated cell‐free engineering of cell‐derived EVs or EV mimetics. Indeed, further convergences between EV research and cell‐free synthetic biology may ultimately accelerate future innovations in EV‐based diagnostics, therapeutics, and drug delivery systems for the benefit of patients. More broadly, we foresee an array of opportunities for cell‐free engineered EVs across a broad spectrum of foundational biomedical research and translational healthcare applications (Figure 2).

### 1.1 Extracellular vesicles

EVs are nano‐ or micro‐sized lipid‐bilayer vesicles, derived from many cell types, that contain complex molecular cargoes including membrane proteins, luminal proteins, metabolites, and nucleic acids (Couch et al., 2021). EV research has accelerated in the last several decades, in part, through the recognition that EVs originate from many different organisms and cell types including mammalian cells, plant cells, parasites (e.g., Helminths), bacteria (e.g., outer membrane vesicles [OMVs]), and archaea (Bonsergent et al., 2021; Borup et al., 2022; Liu et al., 2021; Pinedo et al., 2021; Schwechheimer & Kuehn, 2015). EVs are also found in most bio‐fluids including blood (where they were originally described as coagulation factors), urine, saliva, cerebrospinal fluid (CSF), milk and plant apoplastic fluids (Pinedo et al., 2021; Zhou et al., 2020). Proposed EV biological functions are also diverse and include the disposal of cellular waste, intercellular signalling, tissue homeostasis, immunomodulation, and host‐pathogen interactions (Couch et al., 2021; Kalluri & Lebleu, 2020; Schwechheimer & Kuehn, 2015).

Foundational EV research is also leading to translational insights and the identification of molecular changes in EVs that can occur during disease progression. Essentially, EVs are increasingly being examined as an accessible source of biomarkers (e.g., nucleic acids, proteins) for several diseases including cancers, neurological conditions, and infectious diseases (Borup et al., 2022; Kalluri & Lebleu, 2020; Kelwick et al., 2021; Schwechheimer & Kuehn, 2015). As such, studying EVs and their roles in biological processes related to human health and disease is becoming an important area of biomedical research (Kalluri & Lebleu, 2020; Pearson et al., 2021; Schwechheimer & Kuehn, 2015). There is also great potential for EVs, including exosomes, to build upon the progress of liposome‐based drug delivery systems to enable new therapeutic modalities and enhanced targeted delivery of therapeutic cargoes to appropriate organs and tissues (Van Der Koog et al., 2022). EVs isolated from particular cell types (e.g., stem cells) are also directly emerging as a promising new class of nanovesicle therapeutics across oncology, CNS disorders and inflammatory diseases (Kalluri & Lebleu, 2020; Soekmadji et al., 2020). Strikingly, the EV/exosome therapeutic industry is rapidly growing, with industry strategic partnership deals now regularly close to or exceeding $1Bn (USD), with several EV‐based or EV‐enriched therapeutics currently in the clinical pipeline (Rezaie et al., 2022; Zipkin, 2020).

Improvements in exosome metrology and research quality standards (e.g., MISEV and EV‐TRACK) are also helping the field to further refine our understanding of EV heterogeneity, and may lead to the recognition and more precise characterisation of different EV types—whether they be ‘natural’ or created as an experimental artefact (e.g., by cell extrusion, tissue homogenisation or cell‐free engineering) (Lötvall et al., 2014; Pinedo et al., 2021; Sato et al., 2016; Théry et al., 2018; Van Deun et al., 2017). The use of appropriate and consistent EV‐terminology when reporting EV studies is, therefore, vital to ensure research robustness and clarity. As such, the International Society of Extracellular Vesicles (ISEV) has endorsed the use of the term ‘extracellular vesicle’ (EV) to denote all cell‐derived particles that have a lipid bilayer and that lack the capacity for replication (i.e., do not contain a functional cell nucleus) (Théry et al., 2018). This broader definition was recommended for general use by the ISEV community to better reflect the fields continually evolving knowledge of extracellular vesicle types, their biogenesis, molecular compositions, and biological functions. However, classical or more specific EV terminology can be used to denote particular EV types where there is sufficient experimental evidence or scientific justification (Lötvall et al., 2014; Théry et al., 2018). For example, the term ‘exosome’ can be used to denote eukaryotic EVs that specifically form through intraluminal budding of endosomal compartment membranes into vesicles that populate the lumen of multi vesicular bodies (MVBs). Subsequent fusion of MVBs with the plasma membrane facilitates exosome release from the cell and their secretion out into the extracellular space (Couch et al., 2021; Zhang et al., 2019) (Figure 2a). Whilst ‘ectosome’, ‘microvesicle’ or ‘apoptotic body’ EVs might simply have shed off from the plasma membrane because of general cellular dynamicity, via cytoskeletal and cell membrane reorganisations, or as part of a physiological process (e.g., apoptotic bodies [ApoBDs] that bleb‐off the cell during programmed cell death [apoptosis]) (Figure 2a) (Kakarla et al., 2020; Zhang et al., 2019). Mechanisms regarding the production of bacterial outer membrane vesicles (OMVs) have also been proposed (Schwechheimer & Kuehn, 2015) (Figure 2a). However, not to complicate the EV nomenclature further, we propose that future cell‐free EV engineering studies remain consistent with MISEV terminology guidance, particularly in relation to the EVs that are used within cell‐free reactions.

### 1.2 Cell‐free synthetic biology

An important aspect of synthetic biology is its focus on utilising a combination of multidisciplinary scientific knowledge and engineering principles to rationally design, build and test biological systems (Brooks & Alper, 2021; Kelwick et al., 2014). Essentially, synthetic biology seeks to re‐purpose existing biological components (e.g., promoters, enzymes, etc.) or create entirely synthetic devices and systems (e.g., biosensors), not just for the goal of creating useful biotechnologies in a responsible manner, but to do so in a way that deepens our fundamental understanding of biology (Brooks & Alper, 2021; Kallergi et al., 2021; Kelwick et al., 2014). Indeed, cell‐free systems utilise isolated cellular components within *in vitro* biochemical assays and were originally conceived as foundational tools to study the underlying mechanisms of cellular biochemistry, molecular biology and protein synthesis (Garenne et al., 2021; Hecht et al., 2017; Kelwick et al., 2014; Silverman et al., 2020). Whilst many different types of cell‐free systems have been developed, CFE and concomitant *in vitro* protein production remain a key application focus (Borkowski et al., 2020; Bundy et al., 2018; Garenne et al., 2021). To this end, CFE systems that make use of an expanding array of microbial (*Escherichia coli, Bacillus subtilis*, *Vibrio natriegens*, etc.) or eukaryotic cell extracts (Chinese Hamster Ovary [CHO] cells, HeLa cells, *Pichia pastoris*, wheat germ, *Leishmania tarentolae*, etc.), have been developed or optimised in recent years (Gagoski et al., 2016; Heide et al., 2021; Kelwick et al., 2016; Spice et al., 2022; Sun et al., 2013; Wiegand et al., 2018). These CFE systems typically utilise the cellular transcription/translation machinery and metabolic pathways (ribosomes, Krebs cycle enzymes, etc.) found within the cell extracts, in combination with optimised reaction mixes (amino acids, energy substrates, enzyme co‐factors, etc.), to enable *in vitro* protein production from engineered linear or plasmid DNA templates (Cai et al., 2015; Guzman‐Chavez et al., 2022; Mcsweeney & Styczynski, 2021; Yim et al., 2020). Entirely reconstituted cell‐free systems, including the protein synthesis using recombinant elements (PURE) system, have also been established (Shimizu et al., 2001).

CFE systems are a useful part of the synthetic biology research toolkit and advances in cell‐free synthetic biology have also enabled new medical, environmental and biotechnological applications (Garenne et al., 2021; Silverman et al., 2020; Zawada et al., 2022). For example, cell‐free‐based biosensors have been developed that can detect bacterial and viral respiratory infections (Karlikow et al., 2022; Pardee et al., 2014; Wen et al., 2017), or a panoply of drinking water contaminants (Jung et al., 2020). Likewise, innovations in cell‐free metabolic engineering (Dudley et al., 2015) and biomanufacturing are enabling significant progress towards the cell‐free‐based manufacturing of therapeutics and vaccines (Ogonah et al., 2017; Pardee et al., 2016). Cell‐free‐based structural biology and drug discovery studies might also lead to future therapeutic interventions (Bruni et al., 2022; Novikova et al., 2018). Cell‐free systems have important advantages that have made these applications possible. For instance, the open nature of cell‐free reactions allows for the direct addition of useful molecular components such as programmable DNA, chaperones, or lipid nanostructures, that can facilitate the production of cytotoxic and/or difficult to express proteins (Bundy et al., 2018; Garenne et al., 2021)—including membrane proteins of relevance to drug discovery and EV biology (Kruyer et al., 2021; Sachse et al., 2013; Shinoda et al., 2016; Umbach et al., 2022). This is also beneficial in that cell‐free systems can, therefore, facilitate reaction conditions and applications that might not be viable in live cells. Moreover, molecular insights from small‐scale prototyping cell‐free reactions have also proven to be somewhat interoperable between CFE (*in vitro*) experiments and live whole‐cells (*in vivo*) (Chappell et al., 2013; Kelwick et al., 2016; Silverman et al., 2020). As such, insights from cell‐free prototyping reactions might later inform cell‐based applications. Beneficially, cell‐free workflows are also compatible with automation platforms and design‐of‐experiment (DOE) approaches, which can help them to be completed much more rapidly than cell‐based assays (Chappell et al., 2013; Kelwick et al., 2020; Kopniczky et al., 2020). Previous studies have shown that cell‐free reaction volumes can also be scaled from nanolitres (nL) (Kopniczky et al., 2020) to hundreds of litres (L) (Zawada et al., 2011), and that this scalability can be readily exploited to produce, at various yields, peptide therapeutics, vaccines, or biologics (Pardee et al., 2016; Silverman et al., 2020; Stark et al., 2021; Zawada et al., 2011). Cell‐free synthetic biology has also made great strides towards a continual improvement in standardising cell‐free protocols to better ensure consistency of reaction batches, whilst also maintaining optimal performance (Katsura et al., 2017; Zawada et al., 2011).

The interfaces between cell‐free synthetic biology and materials (e.g., paper, liposomes, hydrogels, microfluidics, etc.) have also greatly expanded the application spaces of cell‐free systems (Kelwick et al., 2020). For example, microfluidic‐based cell‐free reaction experiments have enabled the parallel characterisation of many biological parts (e.g., proteins or regulatory elements) (Kelwick et al., 2020; Silverman et al., 2020) as well the generation of artificial cells within microfluidic droplets (Li et al., 2022). Such approaches could conceivably be adapted to produce cell‐free engineered EVs containing many different therapeutic cargoes (Figure 2b). Cell‐free systems can also be lyophilised, or silica dried into either pellets or directly interfaced with materials (e.g., paper). This expands the utility of cell‐free gene expression systems and has been posited as a route towards the on‐demand production of cell‐free‐based diagnostics or therapeutics (Bundy et al., 2018; Guzman‐Chavez et al., 2022; Jaenes et al., 2022; Kelwick et al., 2020). Essentially, standardised cell‐free reactions could conceivably be geographically distributed and then reconstituted locally, as required, with a customised genetic programme that encodes an appropriate biologic, vaccine or diagnostic genetic circuit (Ogonah et al., 2017; Pardee et al., 2016; Tinafar et al., 2022). It is conceivable that such approaches could be further developed to also incorporate cell‐derived EVs or EV mimetics, whereby their inclusion might enable the targeted delivery of the cell‐free produced therapeutic or add additional therapeutic modalities (e.g., theragnostics; Figure 2).

Furthermore, whilst we recognise that significant knowledge gaps and technical challenges remain before cell‐free engineered EV therapeutics might be manufactured in this way, from our perspective it is the continued convergence of EV research and cell‐free synthetic biology that might accelerate the required progress. Essentially, it is our view that EV research can immediately benefit from advancements in cell‐free synthetic biology and the advantages that cell‐free reactions provide. Indeed, it is already possible to incorporate cell‐derived EVs or EV mimetics within cell‐free reactions and then use these systems to expose EVs to defined biochemical conditions or to augment them with *in vitro* produced nucleic acids, small molecules, or proteins. We envision that the inclusion of cell‐derived EVs or EV mimetics within cell‐free reactions could be exploited to investigate additional mechanistic aspects of EV biology (e.g., cargo loading), or to rationally prototype or manufacture engineered EVs loaded with defined molecular cargoes, for use in foundational studies or biomedical research (Figure 2). In the longer term these developments might then enable larger‐scale manufacturing of cell‐free engineered EV therapeutics (Figure 2b).

### 1.3 Feasibility of cell‐free EV engineering

In order to demonstrate the feasibility of cell‐free EV engineering, we undertook several proof‐of‐concept experiments (Figure 1; Supplementary Figures and Supplementary materials and methods). Essentially, we designed pilot experiments to assess the cell‐free production, and EV incorporation, of an enhanced green fluorescent protein (EGFP) and tetraspanin (CD63) fusion protein (EGFP‐CD63). EGFP‐CD63 was identified as a suitable candidate for these pilot experiments since CD63‐fusions have been previously established as a viable EV engineering target in cell‐based studies (Curley et al., 2020). Empty vector (pCFE), GFP, and EGFP‐CD63 cell‐free gene expression plasmids were, therefore, sourced and cloned as described in the supplementary materials and methods. To generate EVs for cell‐free gene expression experiments we first cultured HEK293 cells within a hollowfibre cell culture system, and isolated EVs from conditioned cell media, using an ultracentrifugation‐based method (Supplementary materials and methods). Isolated HEK293 EVs were characterised using nanoflow cytometry (Figure 1d,e; Mean nanoparticle size: 68.4 ± 14.5 nm; concentration: 2.38 ± 0.44 × 10^11^ particles/mL) and a nanoparticle tracking analysis (NTA) system (Figure S1; Mean particle size: 155 ± 66.2 nm; concentration: 5.14 ± 1.05 × 10^11^ particles/mL). Isolated HEK293s EVs were also analysed for EV and cellular protein markers using an Exo‐Check dot blot array (Figure S2), through which the EV marker protein ALIX, was strongly detected (Fordjour et al., 2022). A cis‐Golgi matrix protein, GM130, was also detected, suggesting possible minor cellular protein contamination. Furthermore, ExoView Human Tetraspanin Kit and ExoView R100 system analyses confirmed the presence of a panel of tetraspanin markers (CD63/CD81/CD9) in control (pCFE +EVs) and cell‐free engineered HEK293 EVs (EGFP‐CD63 +EVs) (Figure S4). Characterised HEK293 EVs were subsequently used in time course and dialysis‐mode cell‐free gene expression reactions.

We used a HeLa cell‐extract based cell‐free gene expression system due to its previously demonstrated utility as a prototyping platform for mammalian cell‐free synthetic biology (Kopniczky et al., 2020). However, for the present study, we adapted the manufacturer's suggested protocol for cell‐free EV engineering (Supplementary materials and methods). We first used small‐scale (10 μL volume) cell‐free gene expression prototyping reactions within a 384‐well plate. These cell‐free reactions were incubated at 30°C within a CLARIOstar plate reader, and time course fluorescence measurements indicated that GFP and EGFP‐CD63 proteins were produced in appropriate cell‐free reactions, whereas the fluorescence measurements of cell‐free reactions containing an empty vector control plasmid (pCFE) remained low throughout the 10‐h time course (Figure 1a). Importantly, the inclusion of EVs (+EVs) within appropriate cell‐free reactions did not inhibit the cell‐free production of GFP or EGFP‐CD63 proteins (Figure 1a). However, we observed that endpoint (24 h) fluorescent measurements of dialysis‐mode cell‐free reactions containing HEK293 EVs (+EVs), produced lower yields of GFP or EGFP‐CD63 than those without (Figure 1c). Whilst we cannot directly explain these observations, previous studies have shown that EVs from various cell sources, including HEK293 cells, can be a source of metabolites (e.g., amino acids) as well as active metabolic enzymes (e.g., fatty acid oxidation enzymes) and inhibitors of mitochondrial oxidative phosphorylation (Clement et al., 2020; Do Minh et al., 2021; Zhao et al., 2016). Each of these EV‐associated components could be either beneficial or deleterious to cell‐free transcription or translation processes and, if present, may explain our observations (Figure 1c). Future studies could potentially optimise cell‐free EV engineering workflows, through careful characterisation and optimisation of EV‐associated metabolic components and activities. However, for our purposes, cell‐free protein production yields in EV‐containing reactions were more than sufficient for downstream analyses. Indeed, the fluorescence of GFP and EGFP‐CD63 proteins in cell‐free EV engineering reactions was clear enough that it could be directly visualised on a Blue LED transilluminator (Figure 1b).

Semi‐continuous dialysis‐mode cell‐free reactions were subsequently analysed using a nanoflow cytometer which was calibrated, using QC beads and reference silica nanospheres, for size and light scattering detection of EVs (Lees et al., 2022). Nano flow cytometry analysis of cell‐free reactions indicated the presence of nanoparticles/EVs in all samples and, as expected, a low number of particulates were detected in the HEPES/NaCl buffer blank (Figure 1d–f). Gated events in EGFP‐CD63 or EGFP‐CD63 +EV samples indicate strongly green fluorescent (FITC‐A/GFP) nanoparticles/EVs (Figure 1d). We observed that the FITC/GFP signals of these gated nanoparticles/EVs were above sample background levels and were also above the high FITC background levels that we observed in GFP or GFP +EV samples (Figure 1d). Therefore, indicating co‐localisation of cell‐free produced EGFP‐CD63 protein with nanoparticles/EVs. The detection of nanoparticles/EVs in cell‐free reaction samples that were not supplemented with exogenous HEK293 EVs (i.e., pCFE, GFP and EGFP‐CD63; Figure 1d,e) is unsurprising given that previous reports have described the presence of endogenous endoplasmic reticulum (ER)‐derived microsomes and other particulates in cell‐free extracts (Dondapati et al., 2020). Nanoflow cytometry analysis of cell‐free reactions that were not loaded with exogenous HEK293 cell‐derived EVs (e.g., pCFE, GFP or EGFP‐CD63) can potentially be used to assess the presence, size, and concentration of some of the microsomes or other nano particulates that are present in the cell‐free reaction components (i.e., cell extracts). Mean detectable nanoparticle/microsome size in cell‐free reactions that did not contain exogenous HEK293 EVs was ∼99 nm (Figure 1e). The presence of microsomes in cell‐free extracts is an experimental artefact related to the methods used for cell lysis and the preparation of cell‐free gene expression extracts. Therefore, these nano flow cytometry data (gated populations) indicate that cell‐free produced EGFP‐CD63 protein may have integrated into or co‐localised with both endogenous microsome fragments and exogenous HEK293 EVs (Figure 1d–f). An additional consequence of the presence of microsomes within cell extracts is that they can ultimately influence mean nanoparticle/EV size and concentration of analysed cell‐free reaction samples. Therefore, future cell‐free EV engineering studies may need to consider whether upstream processing steps are required to remove endogenous microsomes from cell‐free extracts—though this may affect cell‐free performance. Conversely, the PURE cell‐free system could be utilised which is entirely reconstituted and does not contain microsomal contamination (Shimizu et al., 2001). Alternatively, downstream EV purification steps could be used to isolate cell‐free engineered EVs. For example, we used antibody‐coated Dynabeads to isolate CD81‐positive EVs from cell‐free dialysis‐mode reactions (Figure S3). A subset of EVs that were CD81‐Dynabead isolated from EGFP‐CD63 cell‐free reactions were also GFP positive (fluorescence) when analysed using standard flow cytometry. Thus, indicating co‐localisation of EGFP‐CD63 protein with cell‐free engineered EVs (Figure S3). Additionally, an ExoView Human Tetraspanin kit was used to capture cell‐free engineered EVs (EGFP‐CD63 +EVs) onto chips coated with control (IgG) and tetraspanin (CD63, CD81 & CD9) antibody spot arrays. Captured EVs were subsequently immunostained with fluorescent human tetraspanin antibodies (CD63 [Red Channel], & CD81 [Green Channel]) and analysed using an ExoView R100 system. Whilst the blue channel was used to detect EGFP‐CD63 protein fluorescence. In these assays, we detected the co‐localisation of cell‐free produced EGFP‐CD63 protein with CD81 and CD9 positive HEK293 EVs indicating that cell‐free produced EGFP‐CD63 successfully associated with HEK293 EVs (Figure S4B & S4C).

We also confirmed co‐localisation and correct membrane protein topology of cell‐free produced EGFP‐CD63 within a subset of successfully cell‐free engineered HEK293 EVs using a trypsin protease assay and an anti‐GFP antibody (AF‐647) (Figure S5). The purpose of the trypsin assay was to determine EV membrane protein topology given that correctly membrane integrated EGFP‐CD63 protein would have the EGFP internalised within the EV where it would be protected from the proteolytic activity of exogenously added trypsin. EV internalised EGFP would also not be accessible to the anti‐GFP antibody either (Figure S5A). Interestingly, ∼17 ± 4.7% [P4] of total nanoparticles/EVs from EGFP‐CD63 +EV cell‐free reactions had a positive FITC‐A signal (without trypsin treatment) and were likely co‐localised with EGFP‐CD63 protein (Figure S5E). However, only ∼6 ± 1.2% [P4] of total nanoparticles/EVs had a positive FITC‐A signal, post‐trypsin treatment, and therefore represent the subset of cell‐free engineered EVs that contain EGFP‐CD63 with a correct membrane topology (Figure S5E). Whilst nanoparticles/EVs that had positive signals from both anti‐GFP antibody (AF‐647) and FITC‐A (∼2%–4% [P1]) suggest the presence of EV surface aggregated or incorrectly membrane integrated EGFP‐CD63 protein that is also anti‐GFP antibody accessible (Figure S5E). Whereas nano flow cytometry analysis of control and trypsin‐treated GFP +EV samples confirmed our previous observations (e.g., Figure S3). Namely that there is negligible detection of EV surface aggregated GFP in GFP +EV reactions, as evidenced by the low FITC‐A and anti‐GFP antibody (AF‐647) signals (Figure S5B & S5C). Essentially, these data demonstrate that cell‐free EV engineering is feasible, though challenges remain and there is scope for optimisation. These future optimisations might include changes to cell‐free reaction components (e.g., use of different cell lysates or energy mixes), EV and cell‐free sample processing steps, or the development of engineered membrane or cargo proteins that can better integrate with EVs. Importantly, this study has also demonstrated several assays and EV metrology approaches that could be utilised to help optimise future cell‐free EV engineering workflows.

### 1.4 Potential applications for cell‐free engineered EVs

Cell‐free engineered EVs could be utilised to study many different aspects of EV biogenesis, heterogeneity, and cargo incorporation, as well as to study these aspects in relation to their biological significance in physiological or disease processes. This could include directly incubating EVs in biochemical buffers that reflect different aspects of the cell cytosol or endosomal compartment conditions (e.g., pH and salts), and examining aspects of EV stability or molecular cargo loading. This has already been demonstrated for Y‐Box protein 1 and miRNA loading (Shurtleff et al., 2016) and could be expanded to other components (e.g., metabolites, metals, peptides, etc.) through the easy addition of defined cargo compositions and concentrations to the cell‐free reactions. Equally, the cell‐free gene expression of membrane transport proteins (e.g., ion channels) and their integration within a well characterised EV source could also help accelerate fundamental investigations into EV cargo loading. Plate or microfluidic‐based cell‐free EV prototyping workflows could also be used to generate and test, in parallel, many different therapeutic payloads (e.g., nucleic acids or proteins) or cell targeting scaffolds (e.g., CD63‐cell‐targeting peptide fusions), with EVs from many different natural or synthetic sources (Figure 2a,b). These different cell‐free produced cargoes could be loaded into/onto EVs or EV mimetics through membrane fusion protein binding, Y‐BOX miRNA sorting (Shurtleff et al., 2016), small molecule diffusion or be assisted by sonication and/or extrusion (Chen et al., 2021). Cell‐free systems are also compatible with many different unnatural amino acids, xeno nucleic acids, cytotoxic molecules, and other chemical reagents, which in the longer term could lead to designer EVs or EV mimetics with highly engineered synthetic compositions and bioactivities. If cell‐free engineered EV yields are sufficient for downstream analysis, this could help accelerate the development of EV therapeutics and vaccines. From a foundational perspective, this approach could also be utilised to integrate non‐infective viral, bacterial, or parasitic components within a standardised natural or synthetic EV source (e.g., extruded cell fragments or liposomes). This could recreate more safely, and help study, specific aspects of EV biology related to host‐pathogen interactions or immunomodulatory biofunctions. Equally, EVs engineered with disease biomarkers, whether they are human or from an infectious organism (e.g., bacteria), could serve as EV metrology standards in future medical diagnostics. Similarly to EV mimetic‐based approaches (Welsh et al., 2020), cell‐free engineered EVs could potentially serve as metrology reference standards in experimental assays when assessing EV heterogeneity in other contexts as well.

Future innovations in cell‐free synthetic biology methods that generate large cell quantities for lysate generation (Garenne et al., 2021), may also facilitate the scale‐up of cell‐free EV engineering. Essentially, whilst mammalian cell‐free lysates are typically generated from cells cultured in suspension culture (Heide et al., 2021; Stech et al., 2017) we anticipate that, in the future, the production of cell‐free lysates and the generation of large quantities of EVs (for cell‐free engineering) could be carried out using a combined approach. For example, hollowfibre cell culture systems enable high density mammalian cell culture and continuous EV production (Gobin et al., 2021; Kelwick et al., 2020). Therefore, the mammalian cells used to generate EVs could be later harvested and used to produce cell‐free reaction lysates (Figure 2b [middle panel]). Ultimately, we envision that all of these developments may enable innovative foundational extracellular vesicle research approaches and serve as a basis towards novel EV‐based diagnostic or therapeutic approaches (Figure 2c).

## 2 CONCLUSIONS

Cell‐free synthetic biology and the EV research field are complementary in certain respects and even share fundamental scientific approaches. This includes a focus on improving research quality through the continued development of rigorous biological metrology and progress towards robust, standardised experimental protocols. The goal of which is to build a strong foundation to gain a deeper fundamental understanding and insights into biological processes and mechanisms. This shared approach can have practical implications and benefits for both fields. Indeed, it is already apparent that aspects of exosome biogenesis and molecular cargo loading (e.g., miRNAs) can be studied *in vitro*, and since this approach could be expanded to study other aspects of exosome biology, there is clear scope for shared learning or co‐development of metrology between the two fields. Furthermore, advancements in cell‐free metrology, particularly the precise characterisation of complex molecular mixtures (e.g., cell extracts), could not only help efforts to better understand cell‐free reaction activities, but could also conceivably be applied to better characterise EV molecular heterogeneity. Equally, studies which utilise cell‐free gene expression systems to create minimal cells could also benefit cell‐free EV engineering efforts, since both approaches typically involve *in vitro* protein production and their incorporation within a lipid nanostructure. Methods to exogenously engineer EVs, including those designed to encapsulate therapeutic molecules within EVs by sonication, passive diffusion, freeze‐thaw cycles, electroporation or extrusion (Herrmann et al., 2021; Sato et al., 2016), could also be adopted by the cell‐free synthetic biology community to aid the development of minimal cell mimetics. Existing exogenous EV engineering/loading methods are also highly complementary to cell‐free EV engineering since cell‐free gene expression systems could be used to generate bespoke molecular cargoes that then require loading into EVs. Therapeutic small molecules could also be added to cell‐free reactions and then later co‐loaded into EVs along with cell‐free produced cargoes. Therefore, cell‐free EV engineering could be a means to expand the exogenous EV engineering toolbox and benefit from the advantages and potential future capabilities of cell‐free synthetic biology including those described in this perspective and the literature (Bundy et al., 2018; Garenne et al., 2021; Kelwick et al., 2020; Meyer et al., 2021). An interesting future approach might also be the EV encapsulation of cell‐free reactions and a DNA‐encoded genetic circuit to create ‘smart’ EVs that produce molecular cargoes in response to external cues and therefore function as responsive bio‐functionalised materials (Kelwick et al., 2020; Laohakunakorn et al., 2020) (Figure 2c [right panel]) or as a new type of minimal cell chassis (Garenne et al., 2021; Laohakunakorn et al., 2020). As such, we envision that future cell‐free EV engineering studies could be a focal point through which multidisciplinary learnings, innovations and foundational biological insights can be shared across both fields. Beneficially, the future adoption of cell‐free EV engineering approaches may help both the EV research field and the cell‐free synthetic biology community, to make further positive contributions to our understanding of human health and disease. From our perspective, these developments also provide a pathway for further convergences that might ultimately accelerate future innovations in EV‐based diagnostics, therapeutics, and drug delivery systems for the benefit of human health.

## AUTHOR CONTRIBUTIONS

**Richard J. R. Kelwick**: Conceptualization; Data curation; Formal analysis; Funding acquisition; Investigation; Methodology; Project administration; Resources; Supervision; Validation; Visualization; Writing—original draft; Writing—review & editing. **Alexander J. Webb**: Conceptualization; Data curation; Formal analysis; Funding acquisition; Investigation; Methodology; Project administration; Resources; Supervision; Validation; Visualization; Writing—original draft; Writing—review & editing‐Equal. **Amelie Heliot**: Data curation; Formal analysis; Investigation; Methodology; Writing—review & editing. **Clara Tresserras Segura**: Investigation; Writing—review & editing. Paul S. Freemont: Conceptualization; Data curation; Formal analysis; Funding acquisition; Investigation; Methodology; Project administration; Resources; Supervision; Validation; Visualization; Writing—original draft; Writing—review & editing.

## CONFLICT OF INTEREST STATEMENT

All authors declared that there are no conflicts of interest.

## Supporting information

Supporting Information
