# Supplementary material for: Opportunities to accelerate extracellular vesicle research with cell‐free synthetic biology
Source: J Extracell Biol. 2023 May 18;2(5):e90. doi: 10.1002/jex2.90 (PMC11080881; doi:10.1002/jex2.90)
Supplement: Supplementary file 1 — Supporting Information [file JEX2-2-e90-s001.docx]

**Supplementary:**

**Opportunities to accelerate extracellular vesicle research with cell-free synthetic biology**

**Richard J. R. Kelwick^1#^, Alexander J. Webb^1#^, Amelie Heliot^1^, Clara Tresserras Segura^2^ and Paul S. Freemont^1,3,4^**

*^1^Section of Structural and Synthetic Biology, Department of Infectious Disease, Imperial College London, London, SW7 2AZ, UK.*

*^2^Department of Metabolism, Digestion and Reproduction, Imperial College London, London, SW7 2AZ, UK.*

*^3^The London Biofoundry, Imperial College Translation & Innovation Hub, White City Campus, 80 Wood Lane, London, W12 0BZ, UK.*

*^4^UK Dementia Research Institute Care Research and Technology Centre, Imperial College London, Hammersmith Campus, Du Cane Road, W12 0NN, London, UK*

#Joint first authors

**Correspondence to:** Dr Richard Kelwick and Prof. Paul Freemont, Department of Infectious Disease, Imperial College London, London, SW72AZ, UK. E-mail: [r.kelwick@imperial.ac.uk](mailto:r.kelwick@imperial.ac.uk), ORCID 0000-0002-0054-6893; p.freemont@imperial.ac.uk; ORCID: 0000-0002-5658-8486

**Supplementary Methods**

**Bacterial strains and general growth conditions**

Plasmid constructs and strains used in this study are listed in Supplementary Table 1. *Escherichia coli* JM109 (Promega UK) was used for both cloning and generation of cell-free gene expression plasmids. For plasmid recovery *E. coli* strains were grown in Luria-Bertani (LB) medium supplemented with 100 μg/ml Ampicillin (final concentration) and cultured at 37 °C with shaking (220 rpm).

**Strain and plasmid construction**

Sourced plasmids CD63-pEGFP C2 (Addgene #62964), pT7CFE1-Chis (ThermoFisher Scientific #88860) and pCFE-GFP (ThermoFisher Scientific #88891) were transformed into competent *E. coli* JM109 (Promega) to create strains pRK62, pRK63 and pRK64 respectively (Supplementary Table1).

Plasmid pCFE-EGFP-CD63 (pRK65) was generated to enable cell-free gene expression of *egfp*-*cd63* as follows: firstly, PCR primers CD63pT7_F (RK028) and CD63pT7_R (RK029) were used along with DNA template CD63-pEGFP C2 (pRK62) to generate the *egfp-cd63* PCR product whilst simultaneously integrating flanking *Nde*I and *Not*I restriction enzyme sites. The resultant PCR product was digested with *Dpn*I restriction enzyme to remove any remaining plasmid template (pRK62). Next, the *egfp-cd63* PCR product was digested with *Nde*I and *Not*I restriction enzymes (New England Biolabs, UK), separated on an agarose gel (1% w/v) and the *egfp-cd63* DNA fragment was excised and purified (Zymoclean #D4008, Zymo Research, CA, USA). Secondly, the pT7CFE1-Chis cell-free expression vector (pRK63) was digested with *Nde*I and *Not*I restriction enzymes, separated on an agarose gel (1% w/v) and the digested DNA fragment was excised and purified. Purified vector and insert DNA fragments were ligated (Quick Ligase, New England Biolabs, UK) and transformed into *E. coli* JM109 (Promega) to create strain JM109 pCFE-EGFP-CD63 (pRK65). All oligonucleotide primers for plasmid construction and sequencing are listed in Supplementary Table 2.

For the generation of plasmids for cell-free expression experiments, glycerol stocks of the appropriate strains were used to inoculate 100 ml Luria-Bertani (LB) medium cultures supplemented with 100 μg/ml Ampicillin (final concentration), and these cultures were incubated overnight at 37 °C with shaking (220 rpm). Plasmid DNA was isolated using a Qiagen Plasmid *Plus*Midi Kit (#12943, Qiagen, Germany) and then ethanol precipitated prior to usage within cell-free gene expression (CFE) reactions.

**Hollow fibre cell culture**

HEK293 cells (ATCC) were cultured, as previously described (Kelwick et al., 2021), within a hollow fibre bioreactor (FiberCell Systems, Inc., MD, USA), which was configured with a 20 kDa molecular weight cut off cartridge (#C2011, FiberCell Systems, Inc.) to enable high cell density culture (up to ∼10^9^ HEK293 cells) and the concentration of secreted HEK293 cell products (e.g., >20 kDa proteins and extracellular vesicles [EVs]) within the cartridge. Initially, the hollow fibre cartridge was sequentially primed, pre-culture for 24 h each, by Dulbecco's phosphate-buffered saline (DPBS, 1X; #14190144, ThermoFisher Scientific, MA, USA), then serum-free Fluorobrite Dulbecco's Modified Eagle Medium (DMEM, #A1896702, ThermoFisher Scientific) and finally Fluorobrite DMEM (#A1896702, ThermoFisher Scientific) that was supplemented with 10% Exosome-Depleted Fetal Bovine Serum (FBS, #A25904DG, ThermoFisher Scientific).

Once primed, the cartridge was seeded with 1x10^8^ adherent HEK293 cells. Cell growth rate/viability was indirectly evaluated through daily monitoring of the glucose level of the cell culture medium (#GC001000, FiberCell Systems, Inc.). The cell culture medium was changed once the glucose levels were below half the original level. Once glucose consumption levels significantly increased (e.g., 50% glucose consumed within 24 h) the cell culture media was changed to Fluorobrite DMEM (#A1896702, ThermoFisher Scientific) supplemented with 10% Chemically Defined Medium for High Density Cell Culture (#CDM HD, FiberCell Systems, Inc.). Up to two conditioned media samples (~20 ml volume) were harvested from the hollow fibre cartridge each day.

**Isolation of HEK293 extracellular vesicles**

HEK293 cells were cultured in a hollow fibre bioreactor as described above and 5 ml of harvested conditioned media were used to isolate extracellular vesicles using a previously described ultracentrifugation protocol (Li et al., 2017). Briefly, the conditioned media was first centrifuged at 300*g* for 10 minutes. The supernatant was collected then centrifuged at 2500*g* for 10 minutes. The supernatant was again collected and then further centrifuged at 16500*g* for 30 minutes at 4^o^C with a Thermo Scientific Sorvall wX+ ULTRA SERIES ultracentrifuge and T-647.5 fixed angle rotor. The supernatants were subsequently filtered using a 0.2 μm filter and then centrifuged at 110000*g* for 70 minutes at 4^o^C. The resultant pellet was washed with 0.05 μm filtered DPBS (1X; #14190144, ThermoFisher Scientific), re-suspended in 0.05 μm filtered DPBS and then centrifuged at 110000*g* for 70 minutes at 4^o^C. Post-centrifugation the supernatant was discarded, and the pellet containing extracellular vesicles was re-suspended in 200 μl of 0.05 μm filtered DPBS.

**Nanoparticle tracking analysis (NTA)**

Ultracentrifugation isolated HEK293 cell EVs were diluted (1:2000) within particle-free DPBS (0.2 μm filtered; 1X; #14190144, Thermo Fisher Scientific), gently pipetted for 10 s and then aliquoted into a deep-well, 96-well plate. Samples from the 96-well plate were injected into a NanoSight NS300 NTA instrument equipped with a NanoSight Sample Assistant (Malvern Instruments, UK) autosampler system. The NTA images were recorded and analysed to obtain the concentration and distribution of the sample particles. Additional software version and measurement settings are shown in supplementary figure 1.

**Exo-Check Exosome Antibody Array Kit**

HEK293 cells were cultured using a hollowfibre cell culture system. HEK293-dervived EVs were isolated from cell conditioned media using an ultracentrifugation-based method (see supplementary methods above). Isolated HEK293 EVs were lysed and processed, according to manufacturer’s instructions, and analysed for the presence of a panel of EV markers using the Exo-Check Exosome Antibody Array Kit (#EXORAY200B-4, System Biosciences, LLC., USA). The Exo-Check array included 12 dot blot spots/lines that incorporated antibodies specific for the EV markers CD63, CD81, ALIX, FLOT1, ICAM, EpCam, ANXA5 and TSG101, as well as a cellular contamination control (GM130 cis-Golgi marker) and several assay positive/negative [blank] controls. The dot blot array was developed using Immobilon Crescendo Western HRP substrate (#WBLUR0500, Millipore/Merck, Darmstadt, Germany) and imaged using a ChemiDoc imaging system (Bio-Rad Laboratories Inc., USA).

**Cell-free gene expression reactions**

Cell-free reaction master mixes (100 μl) were prepared using a 1-Step Human High-Yield Mini IVT Kit (ThermoFisher Scientific, #88891) and consisted of the following components: 20 μl Reaction Mix, 10 μl Accessory Proteins, 50 μl HeLa lysate, 4 μg of plasmid DNA (pCFE, GFP or EGFP-CD63), 0 or 5 μl of HEK293 EVs (2.38x10^11^ / ml) and nuclease free water to top up the master mixes to 100 μl. 10 μl portions of these master mixes were aliquoted into individual wells of 384-well plates (Griener bio-one, NC, USA) and measured using a CLARIOstar plate reader (BMG, UK) with the following settings: preheated to 30°C, excitation 483-14 nm, 502.5 dichroic and emission 530–30 nm with 1500 gain. Plates were sealed and incubated within the plate reader at 30°C. Samples were shaken (500 rpm) prior to each 10 min reading cycle during the 10 h time course.

**Semi-continuous dialysis mode cell-free reactions**

Cell-free reaction master mixes (300 μl) were prepared using a 1-Step Human High-Yield Mini IVT Kit (ThermoFisher Scientific, #88891) and consisted of the following components: 60 μl Reaction Mix, 30 μl Accessory Proteins, 150 μl HeLa lysate, 12 μg of plasmid DNA (pCFE, GFP or EGFP-CD63), 0 or 15 μl of HEK293 EVs (2.38x10^11^ / ml) and nuclease free water to top up the master mixes to 300 μl. These master mixes were split into 3x 100 μl replicates and aliquoted into a micro-dialysis cassette (10kDa MWCO, ThermoFisher Scientific/Pierce #88260) that was inserted into a 2 ml microtube pre-filled with 1.4 ml of Dialysis Buffer (from 1-Step Human High-Yield Mini IVT Kit #88891). Cell-free dialysis reactions were incubated in an Eppendorf Thermo Mixer C at 30^o^C with shaking for 24h (950 rpm). Post 24h incubation, these cell-free reactions were pipetted out from the dialysis cassettes and into clean 1.5 ml microtubes. 10 μl of these master mixes were subsequently aliquoted into individual wells of 384-well plates (Griener bio-one, NC, USA) and an endpoint was measured using a CLARIOstar plate reader (BMG, UK) with the following settings: preheated to 30°C, excitation 483-14 nm, 502.5 dichroic and emission 530–30 nm with 1000 gain.

**Nano flow cytometry**

Extracellular vesicle, control cell-free and EV containing cell-free reactions were diluted to either 1:1200 or 1:3600 with 0.2 μm filtered 50 mM HEPES/150 mM NaCl buffer (pH 7.4). Diluted samples were analysed using a nano flow cytometer (Flow NanoAnalyzer, NanoFCM, China). The Flow NanoAnalyzer was calibrated using QC Beads (250 nm SiNP Dual laser QC Beads NanoFCM #QS2503) and size reference standard beads (Silica nanospheres, NanoFCM #S16M-Exo 68-155 nm) were used to calibrate the size distribution of EVs. Samples were recorded for 1 minute and 0.2 μm filtered 50 mM HEPES/150 mM NaCl buffer (pH 7.4) was used as a blank control. Data analysis (including sample thresholding to remove background and detector noise signals) was carried out, according to the manufacturers recommendations and best practices (Lees et al., 2022), using Flow NanoAnalyzer software (v1.10).

**Flow cytometry analysis of immunocaptured extracellular vesicles**

For flow cytometry analysis of controls and immunocaptured extracellular vesicles the samples were setup as follows within 1.5 ml microtubes: 40 μl of Exosome-Human CD81 Dynabeads (#10622D, Thermo Fisher Scientific, MA, USA), 0 or 5 μl of HEK293 EVs (2.38x10^11^ / ml), 0 or 60 μl of appropriate semi-continuous dialysis mode cell-free reaction and 120 μl, 175 μl or 180 μl of 50 mM HEPES/150 mM NaCl buffer (pH 7.4). Samples were pipette mixed and then incubated/mixed at RT for 1 h on a RotoFlex plus mixer with setting ‘oo’ (Argos Technologies/Cole Parmer, IL, USA). Post incubation, samples were transferred to a 96-well microplate (Qiagen, #36985), pipette mixed to resuspend the Dynabeads, and then placed on a magnetic rack (Qiagen, Type A #36915). Supernatants were removed and the samples washed with 100 μl of 50 mM HEPES/150 mM NaCl buffer (pH 7.4) whilst being careful not to lose any CD81 Dynabeads in the process. Samples were washed a further 2 times, as described above, and then finally re-suspended into 120 μl of 50 mM HEPES/150 mM NaCl buffer (pH 7.4) ready for flow cytometry. Samples were measured using an Attune NxT flow cytometer equipped with an auto sampler (ThermoFisher Scientific, MA, USA); BL1-A Excitation 488 nm/Emission 530–30 nm) and data analysis was performed using FlowJo (vX 10.8.1) software.

**ExoView Human Tetraspanin assay and analysis of cell-free engineered HEK293 extracellular vesicles**

The ExoView Human Tetraspanin Kit and assay (#251-1000 NanoView Biosciences, USA / Unchained Labs, CA, USA) was carried out according to the manufacturer’s instructions and made use of supplier provided buffers. Briefly, three replicate control (pCFE +EVs) or EV engineering (EGFP-CD63 +EVs) cell-free reactions were pooled together and then diluted 1:1200 (in 50 mM HEPES/150 mM NaCl buffer [pH 7.4]). These diluted HEK293 EV containing cell-free reactions were further diluted (1:4) by mixing with Incubation Solution II [1X] (12.5 μl diluted cell-free/EV reaction & 37.5μl Incubation Solution II [1X]) and these 50 μl samples (now 1:4800 diluted) were pipetted onto an appropriate (pre-scanned) chip from the ExoView Human Tetraspanin Kit (#251-1000 NanoView Biosciences/Unchained Labs). Sample treated chips were incubated overnight at room temperature to enable EV binding, as appropriate, onto the control (Mouse IgG) or tetraspanin (CD63/CD81/CD9) antibody spot regions. Post incubation the chips were processed according to the manufacturers recommendations and using the components within the Human Tetraspanin Kit. Briefly, post overnight incubation, the chips were washed three times with Solution A I [1X] to remove any sample elements that had not bound to the ship. The chips/samples were then incubated with a cocktail of tetraspanin antibodies (CD63 [Red Channel; CF647], CD81 [Green Channel; CF555] & CD9 [Blue Channel; CF 488A]) made up in Blocking Solution II. Note that the CD9 antibody was excluded for chips treated with EV engineering (EGFP-CD63 +EVs) cell-free reactions so that the blue channel could be utilised to detect for the co-localisation of cell-free produced EGFP-CD63 on cell-free engineered EVs. Antibody labelled chips were subsequently washed several times using Solution A I [1X] and then Solution B I [1X]. Finally, chips were washed with Deionized water and then dried using water surface tension as the chips were carefully removed at a ~45^o^ angle from the DI water. The chips were loaded onto a puck and placed within the ExoView R100 system (NanoView Biosciences/Unchained Labs) for chip scanning and data analysis using the ExoView Analyzer Software (v3.2).

**Trypsin protease treatment assay**

A trypsin protease treatment assay to proteolytically degrade free and EV surface proteins was adapted from a previously published assay (Cvjetkovic et al., 2016). The purpose of the assay is to determine EV membrane protein topology given that proteins inside an EV are protected from the proteolytic activity of exogenously added trypsin protease. Briefly, 42 μl reactions were setup including, 10 μl of dialysis-mode cell-free EV engineering sample (GFP + EVs or EGFP-CD63 + EVs), 30 μl of 50 mM HEPES/150 mM NaCl buffer [pH 7.4] and either 2 μl of Trypsin re-suspension buffer (Promega #V5111) or 2 μl Sequencing Grade Modified Trypsin (Promega #V5111; 20 μg/ml final concentration). These reactions were incubated at 37^o^C for 1 hour in an Eppendorf Thermo Mixer C. Post incubation, reactions were cooled to room temperature (22^o^C), treated with 8 μl cOmplete^™^, Mini, EDTA-free Protease Inhibitor Cocktail (1X, Roche, Germany #11836170001) and incubated at room temperature for 30 minutes to allow for inhibition of trypsin proteolytic activity. Post incubation samples were treated with 0.1 μg of Anti-GFP Alexa Fluor(AF)-647-conjugated antibody (Biotechne, Minneapolis, USA; #FAB42402R-100UG; 2 μg/ml final concentration) and incubated at room temperature (22^o^C) for 30 minutes to allow for antibody binding to accessible green fluorescent protein (GFP). Finally, samples were diluted 1:200 into 50 mM HEPES/150 mM NaCl buffer [pH 7.4] and nanoparticles/EVs were analysed, as described above, using a nano flow cytometer (Flow NanoAnalyzer, NanoFCM).

**Supplementary Table 1. Bacterial strains and constructs**

| **Strain** | **Relevant features** | **Reference(s)** |
| --- | --- | --- |
| JM109 | endA1, recA1, gyrA96, thi, hsdR17 (r_k_^–^, m_k_^+^), relA1, supE44, Δ(lac-proAB), [F´ traD36, proAB, laqI^q^ZΔM15] | Promega UK |
| pRK62 | JM109 CD63-pEGFP C2 [CD63-pEGFP C2]; full length human *cd63* cloned into pEGFP C2 vector for CMV-based mammalian cell expression. | CD63-pEGFP C2 was a gift from Paul Luzio (Addgene plasmid #62964 ; http://n2t.net/addgene:62964 ; RRID:Addgene_62964)  & This study |
| pRK63 | JM109 pT7CFE1-CHis [pCFE]; cell-free expression vector; AmpR | ThermoFisher Scientific #88860 & This study |
| pRK64 | JM109 pCFE-GFP [GFP]; Positive control DNA for cell-free expression of *gfp*; AmpR | ThermoFisher Scientific #88891 & This study |
| pRK65 | JM109 pCFE-EGFP-CD63 [EGFP-CD63]; cell-free expression vector expressing *egfp-cd63*; AmpR | This study |

**Supplementary Table 2. Oligonucleotide primers.**

| **Number** | **Name** | **Sequence** |
| --- | --- | --- |
| **Primers for cloning** | | |
| RK028 | CD63pT7_F | atatcacatatgatggtgagcaagggcg |
| RK029 | CD63pT7_R | tcaatagcggccgcctacatcacctcgtagc |
| **Primers for sequencing** | | |
| RK030 | CMVF | cgcaaatgggcggtaggcgtg |
| RK031 | CD63F | ccgacaaccactacctgagc |
| RK032 | CD63R | acaaaccacaactagaatgcag |
| RK033 | upstreamT7 | aacgacggccagtgaattgtaata |
| RK034 | IRES_REV | atgggtggtggccatattatcatc |
| RK035 | pT7-CFE_SEQ1 | tcctttgaaaaacacgatg |

**
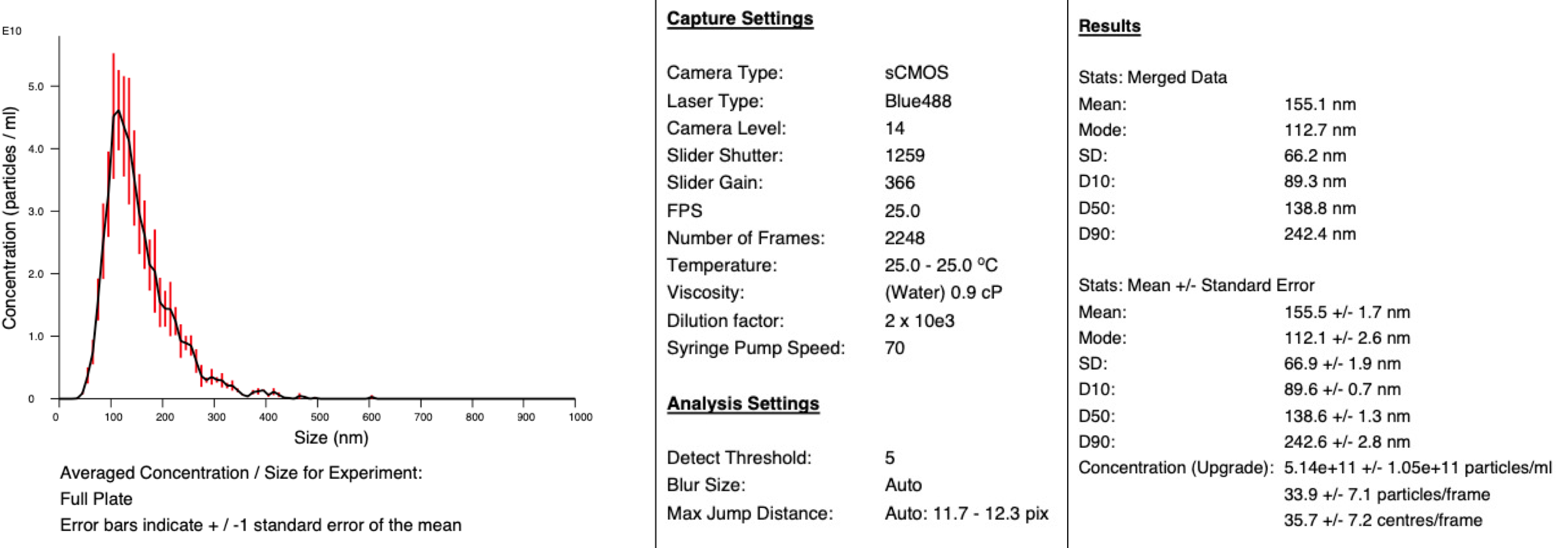
**

**Supplementary Figure 1. Nanoparticle tracking analysis (NTA) of HEK293 extracellular vesicles.** Ultracentrifugation isolated HEK293 cell EVs were diluted 1:2000 into PBS (1X) and analysed using a Malvern NS300 NTA system equipped with a 96-well plate autosampler.

**Supplementary Figure 2. Exo-Check Exosome Antibody Array analysis of HEK293 cell-derived extracellular vesicles.** Ultracentrifugation isolated HEK293 EVs were processed and analysed for the presence of EV markers using the Exo-Check Exosome Antibody Array Kit. The Exo-Check array included 12 dot blot spots/lines that incorporated antibodies specific for the EV markers CD63, CD81, ALIX, FLOT1, ICAM, EpCam, ANXA5 and TSG101, as well as a cellular contamination control (GM130 cis-Golgi marker) and several assay positive/negative [blank] controls. The developed dot blot array was imaged using a Bio-Rad ChemiDoc imaging system.

**
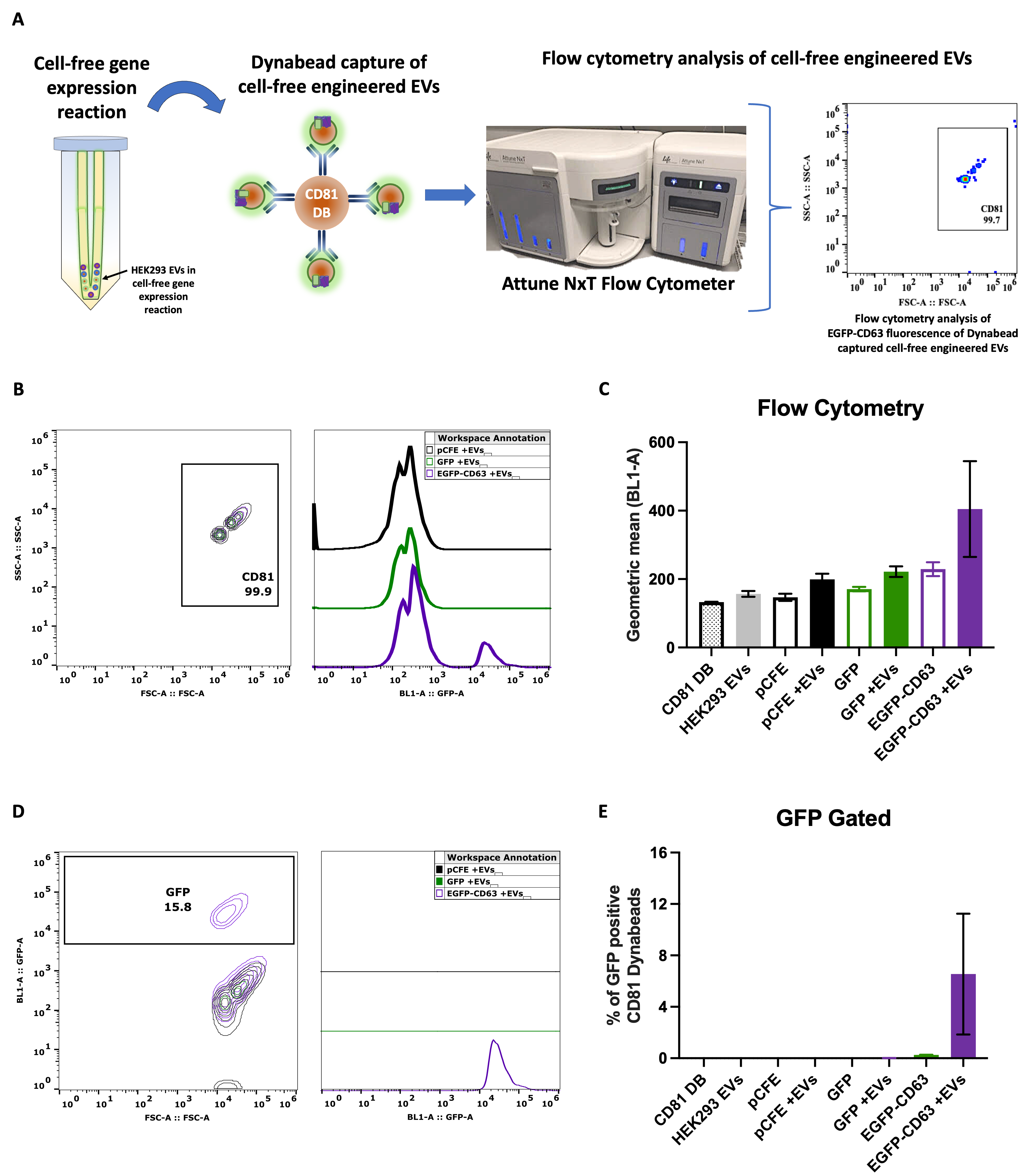
**

**Supplementary Figure 3. Flow cytometry analysis of CD81+ immunocaptured cell-free engineered extracellular vesicles. (A)** Graphical representation of cell-free engineered extracellular vesicles, their immunocapture (CD81 Dynabeads [CD81 DB]) and analysis on an Attune Nxt flow cytometer. **(B)** Representative contour plot (FSC-A/SSC-A), with indicated gating strategy (CD81), and representative fluorescence histograms (BL1-A; GFP-A) of CD81 Dynabead immunocaptured EVs from the indicated cell-free reactions. **(C)** Average geometric means of fresh CD81 Dynabeads (CD81 DB), or CD81 Dynabeads post-incubation with HEK293 EVs or the indicated cell-free reactions. Error bars denote standard error of the mean, n=3. **(D)** Representative contour plot (FSC-A/BL1-A), with indicated gating strategy (GFP), and representative fluorescence histograms (BL1-A; GFP-A) of CD81 Dynabead immunocaptured EVs from the indicated cell-free reactions. **(E)** Average percentage of GFP gated Dynabeads, as a proportion of total Dynabeads per sample. Error bars denote standard error of the mean, n=3 independent dialysis-mode cell-free reactions, ~3,000-5,000 events analysed per sample.

**
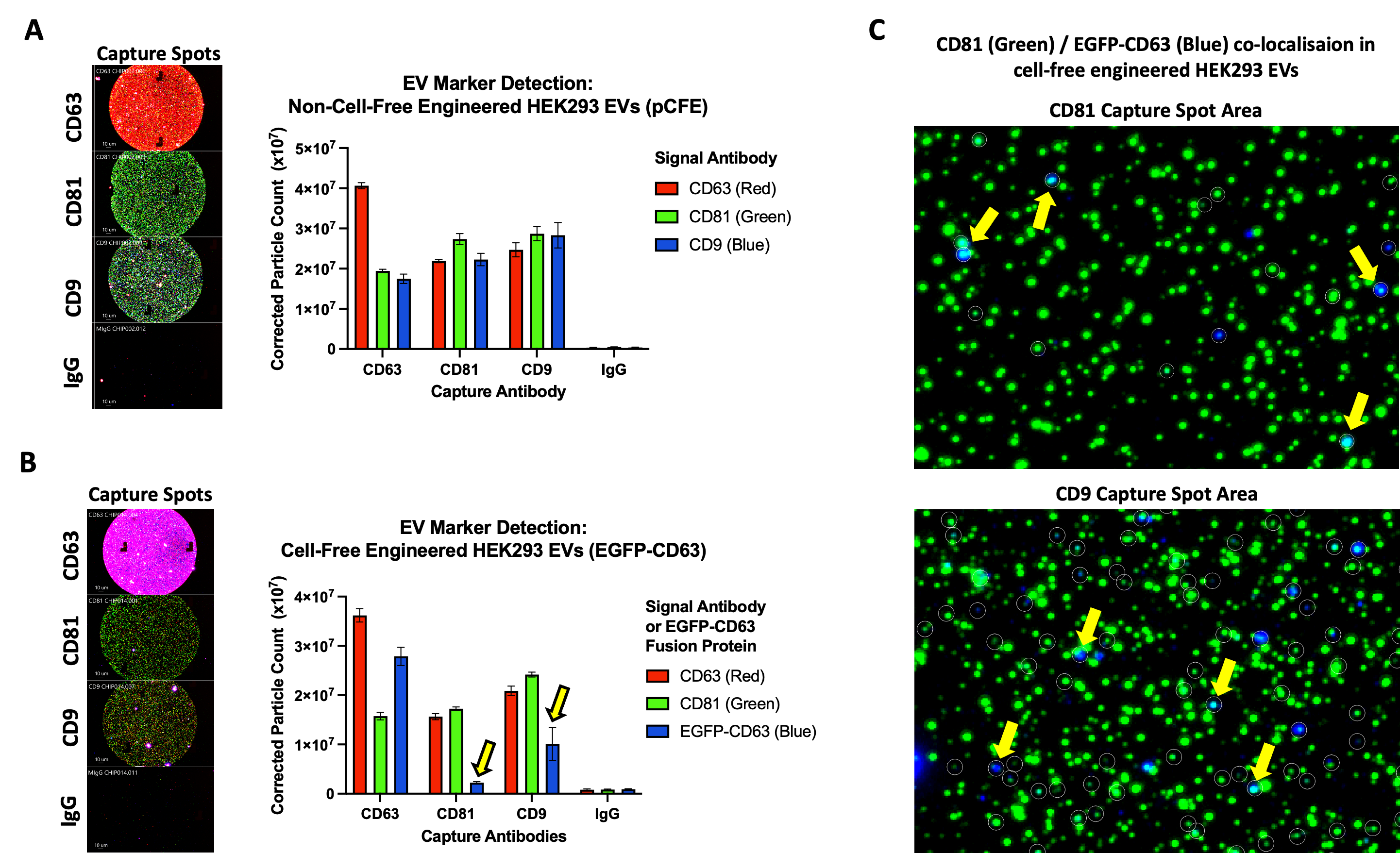
**

**Supplementary Figure 4. Analysis of tetraspanin EV marker and EGFP-CD63 co-localisation in cell-free engineered HEK293 extracellular vesicles.** The ExoView Human Tetraspanin assay involves the capture of EVs onto chips coated with control (IgG) and tetraspanin (CD63, CD81 & CD9) antibody spot arrays. Captured EVs are subsequently immunostained with a cocktail of fluorescent human tetraspanin antibodies (CD63 [Red Channel], CD81 [Green Channel] & CD9 [Blue Channel]) and imaged/analysed using an ExoView R100 system (NanoView Biosciences / Unchained Labs) and ExoView Analyzer Software (v3.2). Representative antibody array spot images are shown in panels (A-C). These assays enable the detection and co-localisation analysis of tetraspanin markers or fluorescent proteins in EV samples. **(A)** Tetraspanin EV marker analysis of non-cell-free engineered HEK293 EVs (pCFE +EVs). Sample dilution corrected fluorescent particle counts of spot array captured and fluorescent tetraspanin antibody (CD63/CD81/CD9) labelled HEK293 EVs from control cell-free gene expression reactions (pCFE) in which the HEK293 EVs were not cell-free engineered. Three replicate cell-free reaction samples (pCFE +EVs) were pooled together and analysed on a single microarray chip; error bars denote standard error of the mean. **(B)** Tetraspanin EV marker and EGFP-CD63 fusion protein co-localisation analysis of cell-free engineered HEK293 EVs (EGFP-CD63). CD9 fluorescent antibody was omitted for these samples so that the blue channel could be utilised to detect for the co-localisation of cell-free produced EGFP-CD63 with cell-free engineered HEK293 EVs. Sample dilution corrected fluorescent particle counts of spot array captured, fluorescent tetraspanin antibody (CD63/CD81) labelled and EGFP-CD63 positive HEK293 EVs from cell-free engineering reactions (EGFP-CD63 +EVs). Yellow arrows on the graph indicate examples of CD81/EGFP-CD63 or CD9/EGFP-CD63 co-localisation on cell-free engineered HEK293 EVs. Three replicate cell-free reaction samples (EGFP-CD63 +EVs) were pooled together and analysed using two separate microarray chips: error bars denote standard error of the mean. **(C)** Images show co-localisation of CD81 (fluorescent antibody [green channel]) and EGFP-CD63 (fusion protein [Blue channel]) on CD81 or CD9 antibody spot captured, cell-free engineered HEK293 EVs (EGFP-CD63). The indicated rings in the images were added automatically by the ExoView Analyzer software to indicate CD81(green)/EGFP-CD63(blue) co-localisation in HEK293 EVs. Yellow arrows were manually added to indicate select examples of CD81/EGFP-CD63 or CD9/CD81/EGFP-CD63 co-localisation on cell-free engineered HEK293 EVs.

**
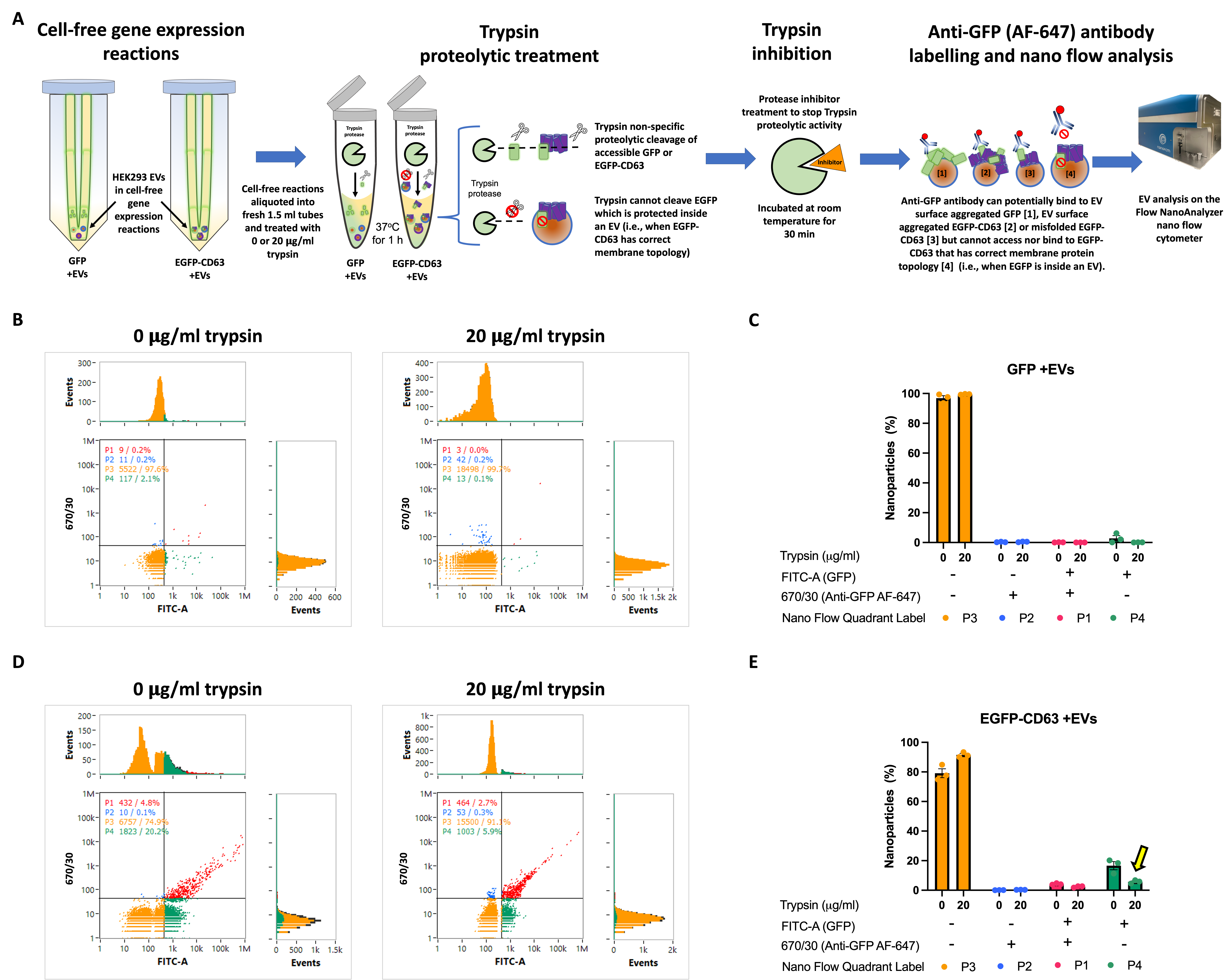
**

**Supplementary Figure 5: Trypsin protease treatment assay to determine EGFP-CD63 membrane protein topology in cell-free engineered EVs. (A)** Trypsin protease assay workflow of [GFP +EVs] and [EGFP-CD63 +EVs] cell-free reaction samples. **(B)** Representative nano flow cytometry scatter plots of HEK293 EVs in GFP producing cell-free reactions [GFP +EVs] that were sequentially treated with 0 or 20 μg/ml trypsin protease, EDTA-free protease inhibitor cocktail and 2 μg/ml anti-GFP Alexa Fluor (AF)-647 antibody. A quadrant gating strategy (P1-P4) was used to identify nanoparticle/EV subpopulations based upon their FITC-A (GFP or EGFP-CD63 detection) and 670/30 nm (anti-GFP AF-647 antibody detection) signals. **(C)** Percentage (%) of total analysed nanoparticles/EVs in [GFP +EVs] cell-free reactions within each of the gated quadrants P3 (FITC -/- Anti-GFP AF-647), P2 (FITC -/+ Anti-GFP AF-647), P1 (FITC +/+ Anti-GFP AF-647) and P4 (FITC +/- Anti-GFP AF-647). **(D)** Representative nano flow cytometry scatter plots of HEK293 EVs in EGFP-CD63 producing cell-free reactions [EGFP-CD63 +EVs] that were sequentially treated with 0 or 20 μg/ml trypsin protease, EDTA-free protease inhibitor cocktail and 2 μg /ml anti-GFP Alexa Fluor (AF)-647 antibody. A quadrant gating strategy (P1-P4) was used to identify nanoparticle/EV subpopulations based upon their FITC-A (GFP or EGFP-CD63 detection) and 670/30 nm (anti-GFP AF-647 antibody detection) signals. **(E)** Percentage (%) of total analysed nanoparticles/EVs in [EGFP-CD63 +EVs] cell-free reactions within each of the gated quadrants P3 (FITC -/- Anti-GFP AF-647), P2 (FITC -/+ Anti-GFP AF-647), P1 (FITC +/+ Anti-GFP AF-647) and P4 (FITC +/- Anti-GFP AF-647). The yellow arrow on the graph indicates examples of HEK293 EVs that have incorporated EGFP-CD63 membrane fusion proteins that have a correct membrane protein topology. Error bars denote standard error of the mean, n=3 independent dialysis-mode cell-free reactions.

**Supplementary References**

Cvjetkovic, A., Jang, S. C., Konečná, B., Höög, J. L., Sihlbom, C., Lässer, C., et al. (2016). Detailed Analysis of Protein Topology of Extracellular Vesicles–Evidence of Unconventional Membrane Protein Orientation. *Sci. Rep.* 6, 36338. doi:10.1038/srep36338.

Kelwick, R. J. R., Webb, A. J., Wang, Y., Heliot, A., Allan, F., Emery, A. M., et al. (2021). AL-PHA beads: Bioplastic-based protease biosensors for global health applications. *Mater. Today* 47, 25–37. doi:10.1016/j.mattod.2021.02.018.

Lees, R., Tempest, R., Law, A., Aubert, D., Davies, O. G., Williams, S., et al. (2022). Single Extracellular Vesicle Transmembrane Protein Characterization by Nano-Flow Cytometry. *J. Vis. Exp.* doi:10.3791/64020.

Li, P., Kaslan, M., Lee, S. H., Yao, J., and Gao, Z. (2017). Progress in Exosome Isolation Techniques. *Theranostics* 7, 789–804. doi:10.7150/thno.18133.
